# 

**Published:** 1905-04-22

**Authors:** 


### NOTICE TO CORRESPONDENTS.

All MSS., Letters, Books for Review, and other matters intended for the Editor should be addressed The Editor, "The Hospital," The Hospital Buildings, 28 & 29 Southampton Street, Strand, W.C.

The Editor cannot undertake to return rejected MS., even when accompanied by stamped directed envelope.

### Notice to Advertisers and Subscribers.

All Advertisements, Orders for Copies of The Hospital, and Business Communications should be addressed to **THE MANAGER (Not to the Editor),** The Hospital, 28 & 29 Southampton Street, Strand, London, W.C.

The Cost of Subscription, post free, is at follows :—Per week, 3½d.; per quarter, 3s. 3d.; per half-year, 6s. 6d.; per annum, 13s. Foreign and Colonial :—Per annum, 19s. 6d., payable, in all cases, in advance.

### Medical Appointments.

**Captain Francis E. Baines**, I.M.S., L.R.C.P.Edin., Clinical Assistant to the Chelsea Hospital for Women. **John P. Ferguson**, L.R.C.P.&S.Edin., L.F.P.S.Glas., Medical Officer of Health of the Misterton Rural District. **J. R. Higson**, M.B., C.M.Edin., Certifying Surgeon under the Factory and Workshop Act for the Lewisham District of the county of London. **A. W. D. Hunt**, M.R.C.S., L.R.C.P.Lond., Certifying Surgeon under the Factory and Workshop Act for the Chagford District of the county of Devon. **S. Kay**, M.B.Syd., Resident Medical Officer at the Sydney Hospital, Australia. **Charles R. Keyser**, F.R.C.S.Eng., Assistant Surgeon to the Cancer Hospital, Fulham Road, S.W. **Samuel J. J. Kirby**, M.R.C.S.Eng., L.R.C.P. Edin., reappointed permanently Medical Officer of Health to the Oulton Board Urban District Council. **Hugh Lett**, M.B., Ch.B.Vict., F.R.C.S., Assistant Surgeon to the London Hospital. **F. S. Mackenzie**, M.B., Ch.B.Edin., House Surgeon to the County Hospital, York. **Stephen Mayou**, F.R.C.S.Eng., Ophthalmic Surgeon to the Hospital for Epilepsy and Paralysis, Maida Vale, London, W. **V. McDowell**, M.B.Syd., Resident Medical Officer at the Royal Prince Alfred Hospital, Sydney, Australia. **J. M. McEncroe**, M.B.Syd., Resident Medical Officer at the Royal Prince Alfred Hospital, Sydney, Australia. **J. L. McKelvey**, M.B.Syd., Resident Medical Officer at the Royal Prince Alfred Hospital, Sydney, Australia. **J. A. Mills**, B.A., M.B., B.Ch., B.A.O.R.U.I., Assistant Medical Officer and Pathologist to the Durham County Asylum. **R. O. Moon**, M.D.Oxon., M.R.C.P.Lond., Physician to Out-patients at the Royal Waterloo Hospital. **R. F. Moore**, M.B., B.C.Cantab., Ophthalmic House Surgeon to St. Bartholomew's Hospital. **A. R. Neligan**, M.B.Lond., Intern Midwifery Assistant at St. Bartholomew's Hospital. **Herbert F. Parker**, M.D.Cantab., M.R.C.S., L.R.C.P., Honorary Assistant Medical Officer to the Royal Surrey County Hospital, Guildford. **James Roland Payne**, M.R.C.S.Eng., L.R.C.P., L.M.Edin., Medical Officer of Health for the Coleford (Gloucestershire) Urban District Council. **G. A. Phillips**, M.R.C.S.Eng., L.S.A., Certifying Surgeon under the Factory and Workshop Act for the Walsall District of the county of Stafford. **C. T. M. Plowright**, B.A., M.B., B.C. Cantab., Senior House Surgeon to St. Bartholomew's Hospital. **J. W. Power**, M.B.Syd., Resident Medical Officer at the Sydney Hospital, Australia. **Ernest H. Price**, L.S.A., Assistant House Surgeon to the Northampton General Hospital. **Charles Randolph**, M.R.C.S.Eng., L.R.C.P., L.M.Edin., Medical Officer of Health by the Wellington (Somerset) Rural District Council. **B. B. Riviere**, M.R.C.S., L.R.C.P.Lond., Junior House Surgeon to St. Bartholomew's Hospital. **A. S. C. Roberts**, M.B.Syd., Resident Medical Officer at the Royal Prince Alfred Hospital, Sydney, Australia. **G. W. Simpson**, M.B.Syd., Resident Medical Officer at the Sydney Hospital, Australia. **J. G. Slade**, M.A., M.B., B.C.Cantab., Junior House Physician to St. Bartholomew's Hospital. **P. E. W. Smith**, M.B.Syd., Resident Medical Officer at the Royal Prince Alfred Hospital, Sydney, Australia. **W. Gordon Speers**, M.R.C.S., L.R.C.P.Lond., Assistant Medical Officer of the Hospital Samaritano, Sao Paulo, Brazil. **A. Verge**, M.B.Syd., Resident Medical Officer at the Sydney Hospital, Australia. **G. H. Vernon**, M.B.Syd., Resident Medical Officer at the Royal Prince Alfred Hospital, Sydney, Australia. **Ernest M. Watkins**, M.B., C.M. Glasg., House Surgeon of Wrexham Infirmary. **E. H. White**, B.A., B.M., B.Ch.Oxon., Senior House Physician to St. Bartholomew's Hospital. **J. K. Willis**, B.A.Cantab., Junior House Physician to St. Bartholomew's Hospital.

### NURSING LITERATURE.

**A HANDBOOK FOR NURSES.**
By J. K. Watson, M.D., M.R., C.M.
**5s.** net, **5s. 4d.** post free.

**NURSING: Its Theory and Practice.**
Medical, Surgical, and Monthly Nursing.
By Percy G. Lewis, M.D. **3s. 6d.** post free.

**OPHTHALMIC NURSING.**
By Sydney Stephenson, M.B., F.R.C.S.E.
With a list of Instruments required, and a Glossary of Terms.
**3s. 6d.** net, **3s. 10d.** post free.

**ELEMENTARY ANATOMY AND SURGERY FOR NURSES.**
By William McAdam Eccles, F.R.C.S.
Profusely illustrated. **2s. 6d.** post free.

**ELEMENTARY PHYSIOLOGY FOR NURSES.**
By C. F. Marshall, M.D., B.Sc., F.R.C.S.
Illustrated. **2s.** post free.

**NURSING IN DISEASES OF THE THROAT, NOSE, AND EAR.**
By P. MacLeod Yearsley, F.R.C.S.
Illustrated. **2s. 6d.** post free.

**HOSPITAL SISTERS AND THEIR DUTIES.**

By Eva C. E. Lückes, Matron, London Hospital. **2s. 6d.** post free.

**OUTLINES OF INSANITY.**
By Francis H. Walmsley, M.D.
**1s. 6d.** post free.

**A COMPLETE HANDBOOK OF MIDWIFERY.**
By J. K. Watson, M.D.Edin.
With 143 Illustrations.
**6s.** net, **6s. 4d.** post free.

**A PRACTICAL HANDBOOK OF MIDWIFERY.**
By Francis W. Haultain, M.D.
Profusely illustrated.
**6s.** net, **6s. 3d.** post free.

**PRACTICAL GUIDE TO SURGICAL BANDAGING AND DRESSINGS.**
By Wm. Johnson Smith, F.R.C.S.
Profusely illustrated. **2s.** post free.

**THE EXAMINATION OF THE URINE.**

By J. K. Watson, M.D.

**1s.** net, **1s. 1d.** post free.

**ART OF FEEDING THE INVALID.**

By a Medical Practitioner and a Lady Professor of Cookery.

**1s. 6d.** post free.

**THE NURSE'S PRONOUNCING DICTIONARY OF MEDICAL TERMS AND NURSING TREATMENT.** By Honnor Morten.
Fifth Edition, with phonetic pronunciation, Edited and Revised by Mary I. Burdett.
**2s.** post free.

**THE HUMAN BODY.**
Its Personal Hygiene and Practical Physiology.
By B. P. Colton.
Profusely illustrated. **5s.** post free.

**ART OF MASSAGE.**
By Mrs. Creighton Hale. **6s.** post free.

### "The NURSING MIRROR"

### "The Nurse's Own Paper."

**Price One Penny weekly. Of all Booksellers and Newsagents.**

NEW CATALOGUE OF NURSING MANUALS POST FREE ON APPLICATION.

*The above Works can be obtained of all Booksellers, or of*

**The Scientific Press, Ltd.,** 28 & 29 SOUTHAMPTON STREET, STRAND, LONDON, W.C.

